# In vivo real-time dynamics of ATP and ROS production in axonal mitochondria show decoupling in mouse models of peripheral neuropathies

**DOI:** 10.1186/s40478-019-0740-4

**Published:** 2019-06-11

**Authors:** Gerben van Hameren, Graham Campbell, Marie Deck, Jade Berthelot, Benoit Gautier, Patrice Quintana, Roman Chrast, Nicolas Tricaud

**Affiliations:** 1 Institut des Neurosciences de Montpellier, INSERM U1051, Université de Montpellier, 34091 Montpellier, France; 2 Department of Neuroscience, Karolinska Institutet, 171 77 Stockholm, Sweden; 3 Department of Clinical Neuroscience, Karolinska Institutet, 171 77 Stockholm, Sweden

**Keywords:** Axonal activity, Demyelination, MFN2, Mitochondria, ROS

## Abstract

Mitochondria are critical for the function and maintenance of myelinated axons notably through Adenosine triphosphate (ATP) production. A direct by-product of this ATP production is reactive oxygen species (ROS), which are highly deleterious for neurons. While ATP shortage and ROS levels increase are involved in several neurodegenerative diseases, it is still unclear whether the real-time dynamics of both ATP and ROS production in axonal mitochondria are altered by axonal or demyelinating neuropathies. To answer this question, we imaged and quantified mitochondrial ATP and hydrogen peroxide (H_2_O_2_) in resting or stimulated peripheral nerve myelinated axons in vivo*,* using genetically-encoded fluorescent probes, two-photon time-lapse and CARS imaging. We found that ATP and H_2_O_2_ productions are intrinsically higher in nodes of Ranvier even in resting conditions. Axonal firing increased both ATP and H_2_O_2_ productions but with different dynamics: ROS production peaked shortly and transiently after the stimulation while ATP production increased gradually for a longer period of time. In neuropathic MFN2^R94Q^ mice, mimicking Charcot-Marie-Tooth 2A disease, defective mitochondria failed to upregulate ATP production following axonal activity. However, elevated H_2_O_2_ production was largely sustained. Finally, inducing demyelination with lysophosphatidylcholine resulted in a reduced level of ATP while H_2_O_2_ level soared. Taken together, our results suggest that ATP and ROS productions are decoupled under neuropathic conditions, which may compromise axonal function and integrity.

**Electronic supplementary material:**

The online version of this article (10.1186/s40478-019-0740-4) contains supplementary material, which is available to authorized users.

## Introduction

While the nervous system, and the brain in particular, represents around 2% of the body mass, it consumes up to 20% of the glucose we mobilize every day [37]. This high energy expenditure in the nervous system is firstly due to the synaptic activity that requires high amounts of ATP [37]. A second energy demanding process is the propagation of action potentials (APs) along axons. Indeed, conduction of APs involves ion exchanges through the plasma membrane, first through voltage-gated ion channels to depolarize, then through the sodium-potassium ATPase to repolarize [4, 5] Therefore, the production of ATP in axons is crucial for repeated regeneration of APs. Both in the CNS and in the peripheral nervous system (PNS), myelination significantly reduces the energy cost of AP propagation through the sequestration of the AP firing machinery at the node of Ranvier and axon initial segment [62].

Mitochondria appear to be the main source of cellular ATP and these organelles are abundant in CNS [24, 48] and PNS [81] axons. Some reports indicated that mitochondria were more abundant in the node of Ranvier [53], but this remains controversial [23]. In addition, axonal AP activity and axo-glial junctions regulates the recruitment of mitochondria in the nodal area [55]. Therefore, a well-accepted idea is that axonal AP activity could locally stimulate ATP production in axonal mitochondria [68].

As an intrinsic by-product of ATP production, mitochondria also produce reactive oxygen species (ROS) [31, 50]. The mitochondrial electron transport chain (ETC) and NAPDH oxidases are the major sources of superoxide (O_2_^•−^), a highly reactive type of ROS, within cells [35]. O_2_^•−^ is rapidly converted to H_2_O_2_ by superoxide dismutase (SOD) and H_2_O_2_ is then reduced to H_2_O and O_2_ by mitochondrial glutathione peroxidase (GPx) and cytosolic enzymes [70]. H_2_O_2_ can also react with iron or O_2_^•−^ to form hydroxyl radicals (OH•), which is then reduced by molecular hydrogen to H_2_O [21]. If not reduced by antioxidants, all types of ROS are highly toxic for the cell and in particular for mitochondria, where they are produced. Indeed ROS can directly oxidize and damage DNA, carbohydrates, proteins or lipids [69].

In several neurodegenerative diseases and axonopathies, such as Parkinson’s disease [19], multiple sclerosis (MS) [67] and Charcot-Marie-Tooth diseases (CMT) [54], mitochondrial dysfunctions result in increased levels of ROS. The importance of mitochondrial homeostasis for the normal function of myelinated axons [55, 65] is highlighted by mutations in the *mitofusin 2* (*MFN2*) gene. Indeed, MFN2 has several functions in mitochondria physiology, among which mitochondrial fusion [14] and transport along axons [49], and mutations in its gene result in axonal form of peripheral neuropathy CMT2A in humans and in mice [13, 83]. In particular, MFN2 loss-of-function in axonal mitochondria decreases the expression of oxidative phosphorylation subunits [56], suggesting that *MFN2* mutations also affect ATP and ROS production. Axonal mitochondria dysfunction is as well involved in MS, a chronic inflammatory disease affecting myelinated tracts in the brain [26]. MS is characterized by demyelinated lesions resulting from the loss of the myelin sheath and the progressive form of MS shows successive events of myelin degeneration and regeneration that damage axons [26]. An increase of mitochondrial ROS contributes to progressive MS [12] and a common hypothesis is that demyelinated axons are more demanding in ATP in order to maintain their functions and their integrity [45, 64].

So, several important questions remain unanswered: How different is the production of ATP and ROS in nodal mitochondria versus internodal mitochondria? How does action potential propagation regulate the dynamics of mitochondrial ATP and ROS production? In what way do axonal or demyelinating neuropathies affect ATP and ROS production in mitochondria of peripheral nerve axons?

To answer these questions, we set up an in vivo approach using a H_2_O_2_-sensitive GFP (roGFP-Orp1) [33] and a fluorescent ATP sensor (ATeam) [40] targeted to mitochondria to image and measure the dynamics of ATP and H_2_O_2_ production in mitochondria of myelinated axons of the PNS. Using this approach, we show that both ATP and H_2_O_2_ levels are increased in mitochondria residing in nodes of Ranvier and that nerve stimulation sharply increases ATP and H_2_O_2_ production by mitochondria in few minutes. While MFN2 mutations had no effect on basal levels of ATP and H_2_O_2_ in axons, it prevented the increase of ATP production after nerve stimulation. At the opposite, an increased H_2_O_2_ production was observed. Moreover, pathological demyelination reduced ATP production while enhancing mitochondrial ROS, showing that both neuropathic conditions decoupled the production of ATP from the production of ROS.

## Materials and methods

### Cloning

pLPCX mito-roGFP-Orp1 (Addgene; #64992) was digested with XhoI/ClaI, blunted and cloned into pAAV-MCS (Cell Biolabs, Inc.) under the control of a CMV promoter. Clones were validated by sequencing. Likewise, pcDNA-mito-ATeam (from H. Imamura, Tokyo, Japan) was digested with XhoI/HindIII, blunted and cloned into the CMV promoter controlled pAAV-MCS. Mitochondria-targeting tags were CoxVIII for roGFP-Orp1 and two tandem copies of CoxVIII for mito-ATeam. AAV viral particles were produced at the viral vector production centre at the Centre de Biotecnologia Animal I Teràpia Gènica in Barcelona, Spain or the University of Nantes, France.

### Mouse strains

Either Swiss mice (Janvier, France) or MFN2^R94Q^ mice [13] were used in the performed experiments. Mice were kept in the animal facility of the Institute for Neurosciences of Montpellier in clear plastic boxes and subjected to standard light cycles. All animal experiments were conducted in accordance with the French Institutional and National Regulation CEEA-LR-11032.

### In vivo virus injection in spinal cord

A thin borosilicate glass capillary (Harvard Apparatus, Ref. 30-0016) was pulled with a Vertical Micropipette Puller (Sutter Instruments, P30–682) to form a glass needle. The glass needle was filled with viral solution (1 μl) and a 1 day old mouse is restrained in a position that exposes the lower back. The needle is injected through the skin using a micromanipulator and introduced into the spinal cord. The viral solution was injected over 2 min with short pressure pulses using a Picopump (World Precision Instrument) coupled to a pulse generator. After injection, the injection site is cleaned with betadine solution (Vetoquinol, cat. no. 3042413) for disinfection and the pup is placed back with its mother and littermates.

### Immunohistochemistry

One month after the viral injection, the sciatic nerve was dissected and fixed in Zamboni’s fixative [66] for 10 min at room temperature. After fixation, the dissected sciatic nerves are washed in PBS and incubated in successive glycerol baths (15, 45, 60, 66% in PBS) for 18 to 24 h each before freezing at − 20 °C. The nerves were cut in small pieces in 66% glycerol and the epineurium sheaths removed. Small bundles of fibers were teased in double-distilled water on Superfrost slides and dried for 3 h at room temperature. Some nerves were frozen in O.C.T. Compound (Tissue-Tek, Ref. 4583) and longitudinal sections were cut using a cryostat (Leica Biosystems, CM3050). For immunostaining, the teased fibers or longitudinal sections were incubated for 1 h at room temperature in blocking solution (10% goat serum and 0.3% TritonX100 in PBS). Then, the samples were incubated with TOM20 (FL-145) primary rabbit antibody (1/500, Santa Cruz, Ref. sc-11,415), mouse anti-Myelin Basic Protein (1/500, Merck, Ref. NE1019) or rabbit NF-200 (1/500, Sigma, Ref. N4142) in blocking solution overnight at 4 °C. The next day, the samples were washed in PBS and incubated for 1 h at room temperature with secondary donkey antibodies coupled to Alexa568 (1/1000, Invitrogen, Ref. A10042) or Alexa488 (1/1000, Invitrogen, Ref. A21202). Finally the samples were washed in PBS and mounted in Immu-mount (Thermo Scientific). Images were acquired at room temperature using a 20× or 40× objective, a Zeiss confocal microscope LSM710, and its associated software.

### Imaging of sciatic nerve in living mice

One month after the viral injection, the mice were anesthetized with a constant flow (1.5 l/min) of oxygen + 5% of isoflurane in an anesthesia box (World Precision Instruments, Ref. EZ-B800) for 5 min. Thereafter the anesthesia was maintained with a mask delivering 2% isoflurane at 0.8 l/min. The eyes were protected by eye protection gel (Ocry-gel, TVM, cat. no. 48026 T613/3). Intraperitoneal injection of 0.1 mg/kg buprenorphine was used for pre-surgery analgesia. The mouse was placed in a silicone mold, lying on its belly, shaved on his hind paw and the paws immobilized using small pins. The incision area was disinfected with betadine solution (Vetoquinol, cat. no. 3042413). The skin was cut using scissors, fat tissue removed, the gluteus superficialis and biceps femoris muscles were separated to reveal a cavity crossed by the sciatic nerve, and the sciatic nerve was lifted up using a small spatula. A long plastic strip was placed underneath the sciatic nerve and this strip fixed using magnets. The sciatic nerve was kept in an aqueous environment of either sterile PBS buffer or artificial cerebrospinal fluid (148 mM NaCl, 3 mM KCl, 1.4 mM CaCl_2_•2H_2_O, 0.8 mM MgCl_2_•6H_2_O, 0.2 mM NaPO_4_•H_2_O in sterile H_2_O) to prevent drying. At that point the mouse was placed under the two-photon microscope objective lens, a glass, 12 mm diameter, 0.25 mm thick microscope coverslip put on top of the nerve and a drop of deionized water placed on it to immerse the objective lens (20X, Carl Zeiss Microscopy, LD CApochromat, Ref. 421,887–9970).

### Demyelination procedure

After imaging of the sciatic nerve in vivo for at least 30 min, 5 μl of 1 mg/ml LPC (Sigma, Ref. N4129) was injected directly into the sciatic nerve using a Hamilton syringe (Ref. 80,930) to induce demyelination. Injection of 5 μl PBS is used as a negative control. After injection, the nerve was placed back to its original location in the body. The skin of the incision was realigned together using a blunt scalpel and stapled along the wound with two clips (Fine Science Tools; 12,020–00). The area around the wound was disinfected again with betadine solution. The anesthesia mask was removed and the mouse was monitored until it had woken up. Motor function of the injected and non-injected hindlimb was followed by lifting the animal by the tail. At 1 week after LPC injection, the non-injected hindlimb showed a normal postural reflex characterized by spreading of leg whereas the injected hindlimb showed abnormal reflexes such as tremors, clasping or retracting of the paw. This behavior has been described and linked to deficient myelination in the PNS in previous studies [39]. The severity of abnormal hindlimb function decreased at 2 weeks after LPC injection and no difference in leg function between the injected and non-injected hindlimb could be observed anymore at 3 weeks after LPC injection.

### Saphenous nerve stimulation in living mice

After skin incision and removal of connecting tissue, the saphenous nerve is lifted up and isolated using a plastic strip (Fig. 2a). To stimulate the saphenous nerve, two platinum kapton microelectrodes (World Precision Instruments, PTM23B05KT) were inserted at the posterior side of the plastic strip using a micromanipulator (Fig. 2a). One hook-shaped recording electrode (AD Instruments, MLA 1203) was placed at the anterior side (Fig. 2a). The ground electrode was inserted in the tail of the mouse and the negative electrode in the groin area (Fig. 2b). The mouse was placed under the two-photon microscope (Fig. 2a), a microscope glass coverslip was placed on top of the nerve and the electrodes were connected to a Powerlab 26 T (AD Instruments; ML4856). A drop of deionized water was then placed on top of the microscope glass to immerse the 20× objective lens.

### Two-photon image acquisition

All in vivo images were obtained with a two-photon microscope LSM 7 MP OPO (Zeiss, France) coupled to a dark microscope incubator (L S1 Dark, Zeiss) in which the temperature was maintained at 37 °C (Heating Unit XL S, Zeiss, France). Mitochondria images were acquired by time-lapse recording varying from one image every minute to one image every 5 min during 1 h. Each image is a stack at Maximum Intensity (ZEN software, Zeiss) of 10 scans over 40 μm depth. For ATeam imaging, a single track at 850 nm excitation wavelength is used to obtain both the CFP (em. 475 nm) and Venus (em. 527 nm) image at the same time point. For roGFP imaging, the two images were acquired for each time point using alternating tracks at 940 nm and 800 nm. Change of track was set after each stack. For Coherent Anti-Stokes Raman Scattering (CARS) imaging, 2 synchronized laser lines at excitation wavelengths 836 nm and 1097 nm are used simultaneously thanks to the OPO system. Each scan was acquired with constant laser intensity (20% for 940 nm, 10% for 850 nm, 10% for 800 nm, 15% for 836 nm, 4% 1097 nm) at a 512 × 512 pixel resolution and microscope imaging parameters were maintained over all different regions we imaged. Images, acquired with ZEN software (Zeiss), were saved in .czi format.

### Data and statistical analysis

We used ImageJ software to analyze the relative ATP or H_2_O_2_ levels in mitochondria of peripheral axons. The acquired images for each wavelength were aligned using the Template Matching plugin. We defined a Region of Interest (ROI) encompassing all labeled mitochondria of the same axon and the mean fluorescent intensity on the ROI was measured on both images. These light intensities were then corrected for background light intensity determined as an area within the nerve where no fluorescent signal from the viral probe can be observed. Either the ATeam Venus/CFP ratio or the oxidized roGFP/reduced roGFP ratio was then calculated from the 2 values for mean light intensity.

Statistical significance for the effect of time on probe stability was determined using linear regression. The effects of the positive controls and negative controls on probe validation were determined using Student two-tailed T-tests. The effect of nerve stimulation was determined using one-way ANOVA and Dunnett’s post-hoc tests. In addition, the threshold for a responding axon was set at 20% change in probe fluorescence ratio. These values for MFN2 mutant mice were obtained using MFN2 mutant and control littermates and the experiments were performed in a blinded way (surgery, imaging and image analysis was performed before knowing the mouse genotype). The differences between MFN2 mutants and wild-type mice were determined using either Student two-tailed T-tests or two- way ANOVA with Sidak post-hoc tests. Differences between internodes and node of Ranvier mitochondria were determined using paired two-tailed T-tests. The effect of demyelination was determined using paired two-tailed T-tests.

## Results

### Validation of the mito-roGFP-Orp1 and mito-ATeam probes

We used ATeam, which is a genetically-encoded fluorescence probe based on fluorescence resonance energy transfer (FRET), to detects ATP [40]. This probe consists of the cyan fluorescent protein (CFP) mseCFP, the yellow fluorescent protein (YFP) variant monomeric Venus, both linked at the ε-subunit of the Bacillus subtilis FoF1-ATP synthase. In the presence of ATP, the ε-subunit retracts to bring the two fluorescent proteins close to each other, thereby increasing FRET efficiency. The conformational change is reversible. Relative ATP levels are measured using the Venus/CFP fluorescence ratio.

Redox sensitive GFPs (roGFPs) harbor an engineered dithiol/disulfide switch on their surface, which determines their wavelength of excitation [2]. RoGFP2 has been converted into an H_2_O_2_ specific probes by fusion with the microbial H_2_O_2_ sensor Oxidant Receptor Peroxidase 1 (Orp1) [33]. The reaction is reversible via reduction by cellular thioredoxin (Trx) or GRx/GSH [58]. Relative H_2_O_2_ levels are measured using the ratio of emitted fluorescence lights when excited at 800 nm (oxidized form) or 940 nm (reduced form). All experiments were done in the same imaging conditions and analysis. As both probes are ratiometric, the measured values are independent of the number of mitochondria or of the absolute fluorescence intensity of the probe. Both probes were targeted to the mitochondrial matrix.

**Fig. 1:**
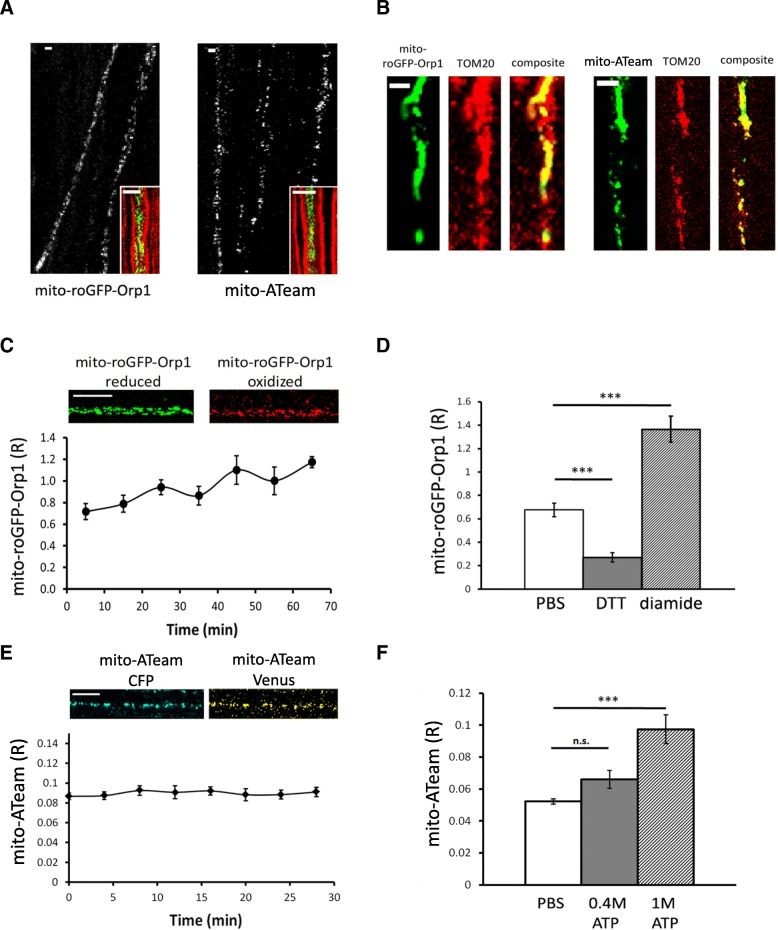
In vivo validation of the fluorescent probes. **a** Several axons expressing mito-roGFP-Orp1 or mito-ATeam probe can be observed in teased fibers of mouse sciatic nerve 1 month after the virus injection. CARS imaging (inserts) shows that axons expressing mito-roGFP-Orp1 or mito-ATeam (green) are surrounded by a myelin sheath (red). Scale bars = 3 μm. **b** Mito-roGFP-Orp1 expression (green) partially colocalizes with mitochondrial marker TOM20 (red) in the axon. Similarly, mito-ATeam partially colocalizes with TOM20 in axonal mitochondria. This partial colocalization is due to the heterogenous distribution of TOM20 in outer mitochondrial membrane [74]. Scale bars = 3 μm. **c** A fluorescent signal of mito-roGFP-Orp1 is detected when the probe is reduced and when it is oxidized by H_2_O_2_. The ratio of mito-roGFP-Orp1 fluorescence measured in vivo (*n* = 6 axons; 6 mice) shows a slight increase over time. Scale bar = 10 μm **d** Mito-roGFP-Orp1 fluorescence ratio decreases with DTT (*p* = 0.006; *n* = 3 axons, 3 mice) and increases after injection of diamide into the nerve (*p* = 0.003; *n* = 4 axons, 4 mice). **e** A fluorescent signal of the CFP subunit and Venus subunit of mito-ATeam is detected. Mito-ATeam fluorescence ratio measured in vivo shows no significant change over time (*n* = 18 axons; 3 mice). Scale bar = 10 μm. **f** Mito-ATeam fluorescence ratio increases after injection of 0.4 M (*p* = 0.06; *n* = 3 axons, 3 mice) and 1 M (*p* = 7.5E-4; *n* = 6 axons, 4 mice) ATP into the sciatic nerve. All error bars show SEM. Statistical analysis shows Student two-tailed T-tests

We first validated mito-roGFP-Orp1 and mito-ATeam probes in vivo. Injection of AAV9-mito-roGFP-Orp1 and AAV9-mito-ATeam in the spinal cord of mouse pups 1 day after birth (P1) resulted in expression of the fluorescent probes in multiple axons that are part of sciatic and saphenous nerves (Fig. 1a). Coherent Anti-stokes Raman Scattering (CARS) microscopy uses the non-linear interactions between light and molecules to generate light from lipids without labeling [72]. As myelin is enriched with lipids, CARS microscopy allows visualizing the myelin sheath around axons in vivo [34, 38, 51]. Using this technique, we observed that the fluorescent probe signal was encompassed by the myelin sheath, which shows the probes are expressed in myelinated axons (Fig. 1a). We then performed an immunostaining of teased fibers for the mitochondrial marker TOM20 and observed a partial colocalisation between fluorescent probes and mitochondria (Fig. 1b), showing probes are expressed in axonal mitochondria, but not all of them. Consistently, mitochondria located in Schwann cells surrounding axons were labeled with TOM20 antibody but not with fluorescent probes. Both probes could be detected in axons of the mouse saphenous and sciatic nerves in vivo.

Then the ratio of the fluorescent intensity of oxidized/reduced roGFP-Orp1 (R) was followed over time (Fig. 1c), showing a small and slow increase of the ratio (0.206 ± 0.002 per hour) probably due to air oxidation. Addition of reducer DTT on the nerve resulted in a significant decrease of the fluorescence ratio, while injection of oxidizer diamide resulted in a significant increase of fluorescence ratio (Fig. 1d). These results show that the dithiol/disulfide switch of mito-roGFP-Orp1 is an efficient and reliable indicator of relative redox changes in axonal mitochondria of mouse peripheral nerves in vivo. The fluorescence ratio Venus/CFP of mito-ATeam (R) was very stable over time (Fig. 1e). Injection of 0.4 M and 1 M ATP into the sciatic nerve in an increase of ATeam fluorescence ratio (Fig. 1f), showing this probe is also functional in vivo.

### The effect of nerve stimulation on ATP and H_2_O_2_ production

**Fig. 2:**
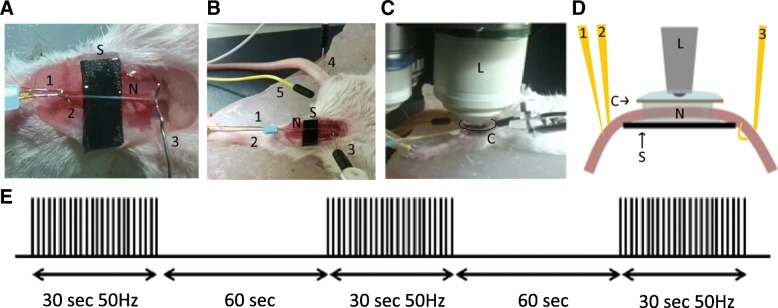
Setup of the saphenous nerve imaging experiment. **a** After deep anesthesia, the mouse is placed on its back, the skin of its left inner tight is removed and the saphenous nerve (N) is placed on a plastic strip (S). Two stimulation microelectrodes (1 and 2) are inserted on both sides of the nerve on the plastic strip and one hook-shaped recording electrode (3) is holding the nerve on the opposite side. **b** The ground electrode (4) is inserted in the mouse tail and the negative electrode (5) in the groin area. **c** A glass coverslip (Co) is then placed on the nerve soaked in PBS and the mouse is then placed under the two-photon microscope immersion lens 20X (L) in water. **d** Schematic view of the imaging setup. **e** Schematic representation of the nerve stimulation pattern used to induce APs. The generation of APs was verified using the recording electrode. Negative electrode was used to correct for background signal

While ATP is supposed to be critical for the firing of axons, ATP production in mitochondria of functionally active axons has never been observed in vivo. We used the real-time imaging approach previously described in combination with a setup for electrical nerve stimulation based on previous work [32, 61] (Fig. 2a-d). We used the saphenous nerve, which is mostly composed of sensory axons, hence its stimulation leads to only limited unwanted contraction of the paw’s muscles [41]. After recording the probe fluorescence for at least 20 min without stimulation, APs were induced by an electrical burst stimulation protocol including 3 bursts of 50 Hz for 30 s spaced by a 60 s recovery period (Fig. 2e).

**Fig. 3:**
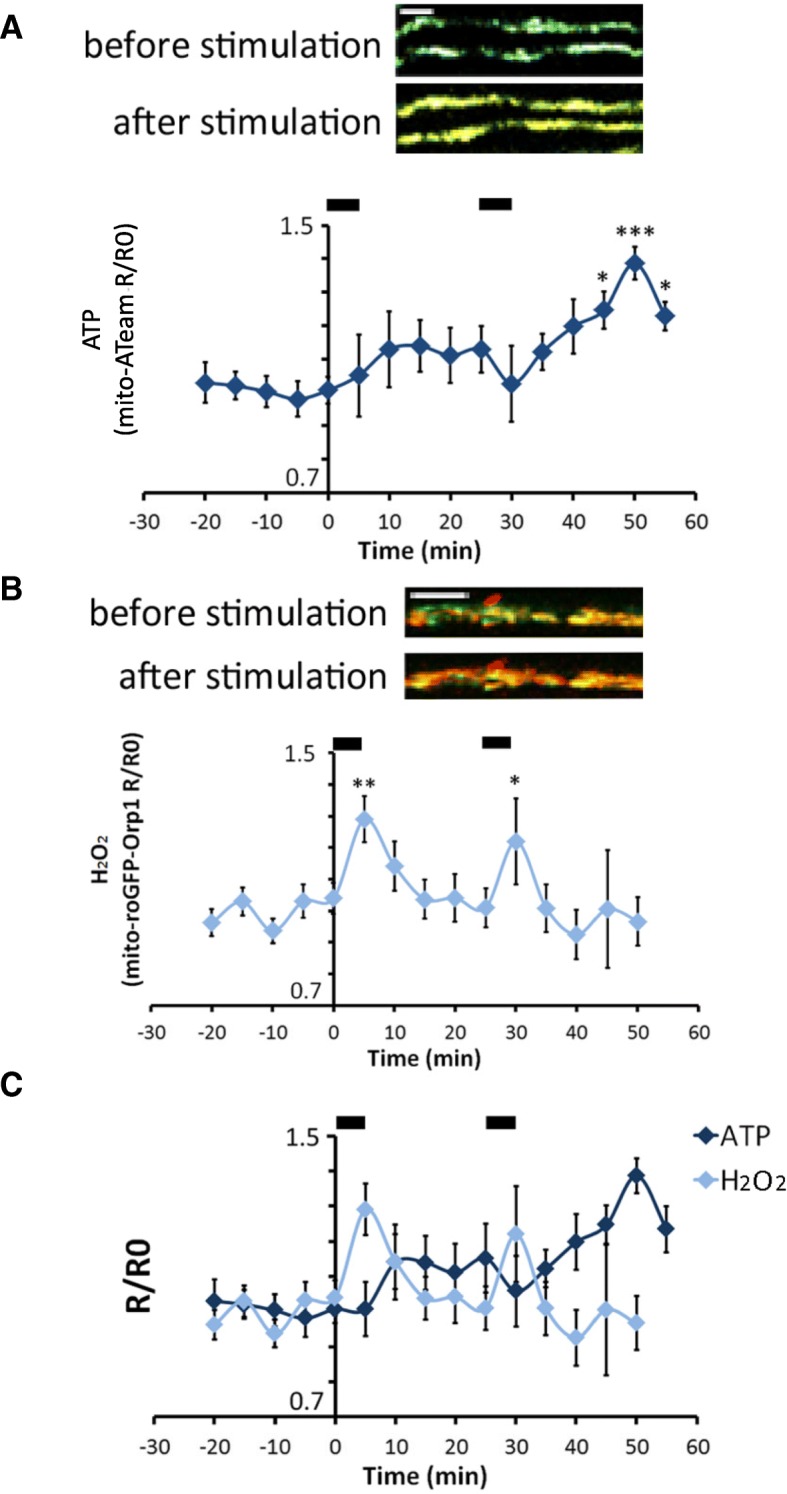
Effect of burst nerve stimulation on mitochondrial ATP and H_2_O_2_ levels. **a** Upper panels: Nerve stimulation induced changes in the fluorescence signal of both the CFP and Venus subunit of mito-ATeam as illustrated by the Venus/CFP overlay pictures. Lower panel: graph showing mito-ATeam fluorescence ratio normalized to pre-stimulation values (R/R0) following two successive nerve stimulation period (black bars at the top). The first stimulation results in a slight, non-significant increase; a significant increase is measured after a second stimulation (*p* = 0.0171 at t = 45, *p* = 0.0001 at t = 50, *p* = 0.049 at t = 55; F-value = 2.872; Df = 15; *n* = 9 axons in 3 mice). **b** Upper panels: Nerve stimulation induced changes in the fluorescence signal of both the oxidized and reduced forms of GFP in mito-roGFP-Orp1 as illustrated by the overlay pictures. Lower panel: graph showing mito-roGFP-Orp1 fluorescence ratio normalized on pre-stimulation values (R/R0) following two successive nerve stimulation (black bars at the top). Both stimulations result in a significant increase 5 min after the stimulation (*p* = 0.007; *p* = 0.04; F-value = 2.804; Df = 14; *n* = 14 axons in 7 mice). **c** Graphs described in a and b were overlaid to show the relative dynamics of ATP and ROS levels after nerve stimulations. Scale bars = 5 μm. Error bars show SEM. Statistical tests are one-way ANOVA

The fluorescent signal of mito-ATeam was recorded every 5 min for 20 min after stimulation (Fig. 3a). At each time point the fluorescence ratio (R) was corrected for the mean fluorescence ratio of that same axon before nerve stimulation (R0). After a first stimulation, the fluorescence ratio of mito-ATeam increased in several axons, but not in all of them (44%), resulting in a slight non-significant average increase (Fig. 3a). After the second stimulation, all axons responded significantly increasing mitochondrial ATP production (Fig. 3a). In the same conditions, nerve stimulations significantly increased mitochondrial H_2_O_2_ production just 1 min after the stimulation period (Fig. 3b). H_2_O_2_ level then quickly went back and stabilized at pre-stimulation values (Fig. 3b). These data show that axonal mitochondria very quickly adapt to the axonal activity up-regulating their production of ATP. Spikes in mitochondrial H_2_O_2_ production were also observed just before the surge of ATP in mitochondria (Fig. 3c), suggesting that this H_2_O_2_ production reflects the oxidative phosphorylation process.

### ATP and H_2_O_2_ production are altered in CMT2A neuropathic mice

**Fig. 4:**
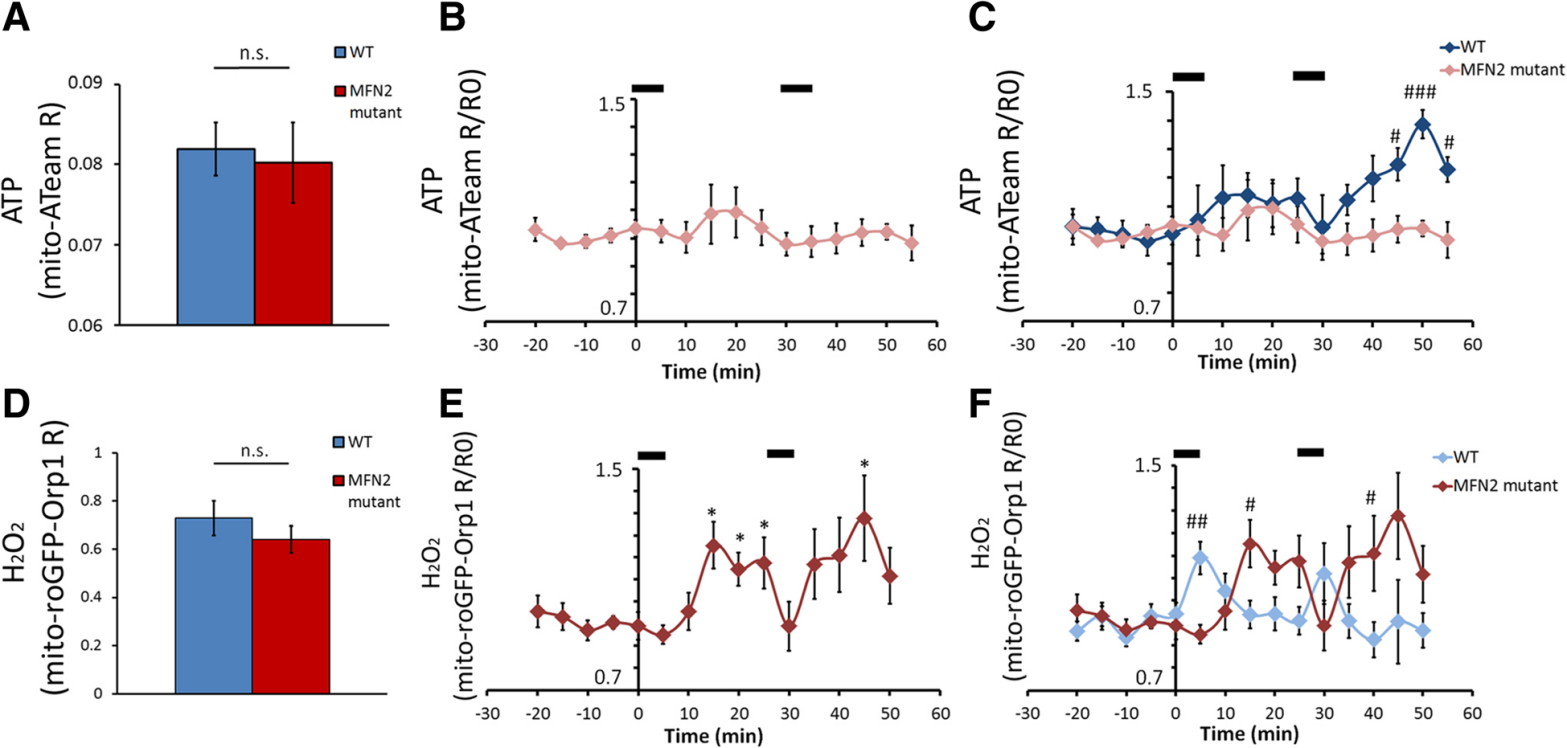
ATP and H_2_O_2_ levels of MFN2^R94Q^ mice. **a** Graph showing mito-ATeam fluorescence ratio in resting axonal mitochondria of wild-type or mutant MFN2^R94Q^ mice (Student two-tailed T-test *p* = 0.809; *n* = 18 axons, 7 mice per group). **b** Graph showing mito-ATeam fluorescence ratio normalized on pre-stimulation values (R/R0) following two successive nerve stimulation periods (black bars at the top). The first period of stimulation resulted in a slight, non-significant increase; the second stimulation period did not generate any change (*n* = 14 axons in 5 mice). **c** Graphs showing mito-ATeam R/R0 in wild-type (Fig. 3a) and mutant MFN2^R94Q^ mice (**b**) were overlaid to show the relative dynamics of ATP levels in both genotypes after nerve stimulations (Two way ANOVA, *p* = 0.011 at t = 45, *p* = 4.8E-5 at t = 50, *p* = 0.02 at t = 55, F-value = 19,27; Df = 1). **d** Graph showing mito-roGFP-Orp1 fluorescence ratio in resting axonal mitochondria of wild-type or mutant MFN2^R94Q^ mice (Student two-tailed T-test *p* = 0.337; *n* = 16 axons, 8 mice per group). **e** Graph showing mito-roGFP-Orp1 fluorescence ratio normalized on pre-stimulation values (R/R0) following two successive nerve stimulation periods (black bars at the top). Both stimulations result in a significant increase (*p* = 0.013 at t = 15, *p* = 0.020 at t = 20, *p* = 0.044 at t = 25 and *p* = 0.038 at t = 45; F-value = 3.095; Df = 14; *n* = 11 axons in 4 mice). **f** Graphs showing mito-roGFP-Orp1 R/R0 in wild-type (Fig. 3b) and mutant MFN2^R94Q^ mice (**e**) were overlaid to show the relative dynamics of H_2_O_2_ levels in both genotypes after nerve stimulations. A significant higher H_2_O_2_ level is detected in wild-type first (Two way ANOVA, *p* = 0.007 at t = 5, F-value = 5484; Df = 1), then a higher H_2_O_2_ level is detected in mutant MFN2^R94Q^ (*p* = 0.014 at t = 15, *p* = 0.047 at t = 40). Error bars show SEM. # = *p*-value< 0.05. ## = p-value< 0.01. ### = p-value< 0.001

We recently showed that MFN2^R94Q^ mice, a model for CMT2A disease [13] where MFN2 is defective, displayed altered mitochondria motility and clustering in peripheral nerve axons [9]. We used this model to measure the level of ATP in axonal mitochondria of myelinated axons in neuropathic conditions. In non-stimulated conditions, control and mutant mice axonal mitochondrial ATP production were similar (Fig. 4a). After the first stimulation, similar to control mice, mitochondrial ATP production increased in some axons whereas others did not respond, resulting in statistically non-significant variation (Fig. 4b). However, after a second period of nerve stimulation, as opposed to controls, MFN2^R94Q^ axonal mitochondria failed to upregulate ATP production (Fig. 4b-c). This indicates that dysfunctional MFN2 does not hinder the basal production of ATP by axonal mitochondria but impairs their ability to up-regulate their production in response to axonal activity.

Similar to ATP, we found no difference between controls and MFN2^R94Q^ mice in the basal H_2_O_2_ levels (Fig. 4d). When nerves were stimulated, mitochondrial H_2_O_2_ significantly increased after each stimulation period (Fig. 4e). However, this increase was delayed compared to controls and H_2_O_2_ levels remained high for longer time (Fig. 4f), showing that axonal mitochondria produce more deleterious H_2_O_2_ than control mice in response to axonal activity. Since ROS production in the mitochondrial matrix reflects the oxidative phosphorylation process [7, 11], these data indicate that oxidative phosphorylation is decoupled from ATP production in mitochondria of these neuropathic mice.

### ATP and H_2_O_2_ production in nodes of Ranvier and internodes

**Fig. 5:**
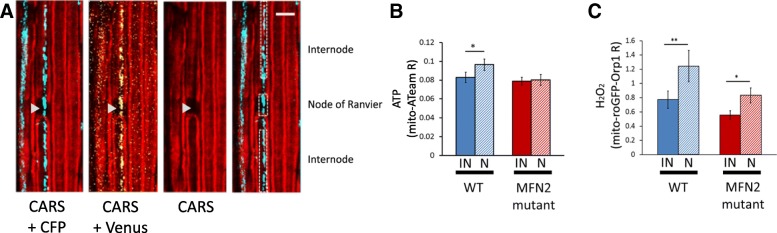
ATP and H_2_O_2_ levels are different along axons. **a** Two-photons imaging of CFP (blue) and Venus (yellow) fluorescence of mito-ATeam was combined with CARS imaging of the myelin sheath (red). CARS imaging gap shows a node of Ranvier (arrowhead). Using the combined images, mitochondria located in the node of Ranvier and mitochondria located in internodes can be identified (right panel). Scale bar = 10 μm **b** Graph showing mito-ATeam fluorescence ratio in axonal mitochondria located in nodes of Ranvier (N) or internodes (IN) of wild-type (WT) or MFN2^R94Q^ mice (MFN2 mutant). In wild-type mice, ATP levels are higher in mitochondria in nodes of Ranvier than in internodes (*p* = 0.035; *n* = 14 axons in 7 mice). In MFN2^R94Q^ mice, ATP levels in node of Ranvier mitochondria are equal to internode mitochondria (*n* = 7 nodes in 3 mice). **c** Graph showing mito-roGFP-Orp1 fluorescence ratio in axonal mitochondria located in nodes of Ranvier or internodes of wild-type or mutant MFN2^R94Q^ mice. In both wild-type mice and mutant MFN2^R94Q^ mice, H_2_O_2_ levels are higher in mitochondria in nodes of Ranvier than in internodes (WT: *p* = 0.009; *n* = 8 axons in 3 mice. MFN2^R94Q^: *p* = 0.019; *n* = 9 axons in 3 mice). Error bars show SEM. Statistical tests are paired two-sided T-tests

The enrichment of mitochondria at the node of Ranvier and whether these nodal mitochondria are more metabolically active remains controversial. In order to detect potential spatial differences between nodal and internodal mitochondria, we used CARS microscopy. Gaps in CARS signal between two myelinated internodes are nodes of Ranvier (Fig. 5a) [8, 11, 38]. Combining two-photon imaging of fluorescent probes with CARS imaging allowed us to analyze mitochondrial physiology in nodes of Ranvier versus internodes defined as areas distant of more than 5 μm from the node (Fig. 5a). In all observed nodes of Ranvier, probes-labeled mitochondria were present, but they were not more abundant or larger than in internodes (Fig. 5a). However, when measuring the mito-ATeam and mito-roGFP-Orp1 fluorescence ratio, we found significantly higher ATP and H_2_O_2_ levels in node of Ranvier mitochondria (Fig. 5b-c). These data, obtained in resting animal without nerve stimulations, indicate that nodal mitochondria are intrinsically more metabolically active than internodal ones. In neuropathic MFN2^R94Q^ mice, nodal mitochondria did not show a higher level of ATP versus internodal mitochondria (Fig. 5b). However, they still had higher level of H_2_O_2_ (Fig. 5c), suggesting again a decoupling between oxidative phosphorylation and the production of ATP in mitochondria of these mutant mice.

### ATP and H_2_O_2_ levels in mitochondria of demyelinated axons

**Fig. 6:**
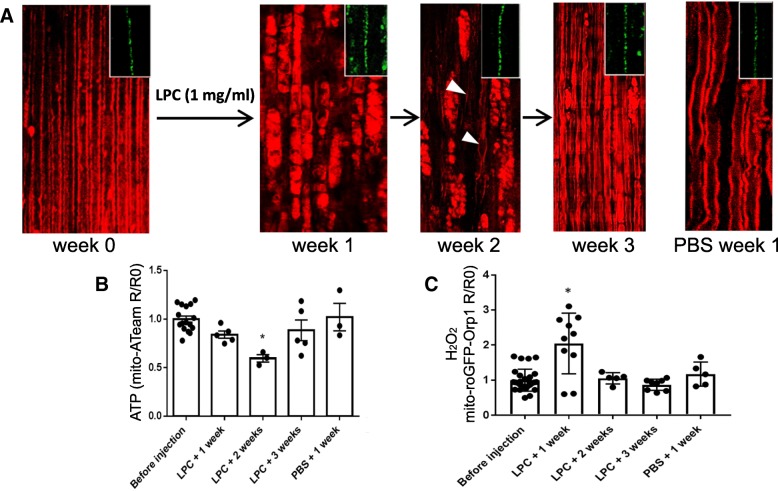
Impact of demyelination on axonal mitochondria ATP and H_2_O_2_. **a** CARS imaging is used to visualize myelin. At week 0, before injection of LPC, most axons are myelinated. 1 week after LPC injection, axons are demyelinated and myelin debris and ovoids are observed. At week 2, thinly myelinated axons are observed in between the myelin debris (arrowheads) and at week 3, almost all axons are myelinated again, thus resembling a healthy nerve. PBS injection induces small conformational changes, but no formation of ovoids or debris. Inserts show that neuronal mitochondria remain visible throughout the whole process. **b** Graph showing mito-ATeam fluorescence ratio (R) in axonal mitochondria normalized on pre-demyelination values (R0) following demyelination. Following LPC injection, ATP levels are unchanged after 1 week (*p* = 0.085; *n* = 5 mice; 27 axons), decreased after 2 weeks (*p* = 0.019; *n* = 3 mice, 24 axons) and restored after 3 weeks (*p* = 0.491; *n* = 5 mice; 31 axons). PBS injection results in no significant change (*p* = 0.799; *n* = 3 mice; 23 axons). **c** Graph showing mito-roGFP-Orp1 fluorescence ratio (R) in axonal mitochondria normalized on pre-demyelination values (R0) following demyelination. Following LPC injection, H_2_O_2_ levels are increased after 1 week (*p* = 0.031; *n* = 10 mice, 17 axons), and unchanged after 2 weeks (*p* = 0.663; *n* = 5 mice, 9 axons) and after 3 weeks (*p* = 0.450; *n* = 8 mice, 13 axons). PBS injection results in no significant change (*p* = 0.121; *n* = 3 mice, 5 axons)

The myelin sheath is critical to maintain the node of Ranvier on myelinated axons and is therefore likely to play a significant role in the homeostasis of axonal mitochondria. As demonstrated in Fig. 5a, the myelination state of the axons in the mouse sciatic nerve can be assessed in vivo by CARS imaging. We therefore investigated how this demyelination affects axonal mitochondria physiology in vivo. To induce demyelination we used lysophosphatidylcholine (LPC) and we followed the levels of mitochondrial ATP and H_2_O_2_ in axons during demyelination and during the restoration of the myelin sheath (remyelination). LPC, which is a signaling molecule resulting from the hydrolysis of phophatidylcholine by phospholipase A2 [17] acts in mSC and oligodendrocytes as a demyelinating signal [3, 42, 57] LPC was injected in the sciatic nerve of adult mice and demyelination occurred immediately to culminate 1 week after the injection with the formation of myelin ovoids and debris as seen using CARS (Fig. 6a, week 1) [34, 71]. As soon as 2 weeks after LPC injection, remyelination occurred and some thin myelin sheaths reappeared around axons (Fig. 6a, week 2). Axons were completely remyelinated 3 weeks after injection of LPC (Fig. 6a, week 3). No demyelination occurred in control mice injected with PBS (Fig. 6a last panel). Axonal integrity is not directly affected by LPC (Additional file 1) [46] and axonal mitochondria remained visible using either mito-ATeam or mito-roGFP-Orp1 probes during the entire process of demyelination and remyelination (Fig. 6a inserts).

We therefore measured the fluorescence ratio for these probes in non-stimulated axonal mitochondria during the process. One week after LPC injection, when axons are completely demyelinated, ATP level was slightly lower than before demyelination, but not significantly (Fig. 6b), while H_2_O_2_ level peaked (Fig. 6c). ATP level was significantly lower in axonal mitochondria at 2 weeks after LPC injection, while H_2_O_2_ level was back to control and pre-demyelination level (Fig. 6b-c). Finally, 3 weeks after injection, when remyelination is completed, ATP an H_2_O_2_ levels were back to control and pre-demyelination levels (Fig. 6b-c). No change either in ATP or H_2_O_2_ levels were detected at 1 week after injection with PBS in control mice (Fig. 6b-c). The increase in ROS did not result from a general oxidative stress in the demyelinated nerve, since CellROX dye did only show a minor fluorescent signal, except at the LPC/PBS injection site where we did not image our probe (Additional file 2). Moreover, the CellROX signal did not show any significant difference between LPC and PBS injected nerves, whereas we did measure differences in mito-roGFP-Orp1 signal. Taken together, these data indicate that the myelin sheath has a profound impact on axonal mitochondria physiology. Moreover, as ATP level decreased during myelin break-down while ROS level increased this suggests that demyelination induces the decoupling of ATP production from oxidative phosphorylation.

## Discussion

Although it has been shown that PNS axonal mitochondria, like most mitochondria, produce both ATP and ROS, the regulations of their production are still not well understood in vivo and in situ. We used a combination of viral delivery of mitochondria-targeted fluorescent probes to active peripheral neurons and two-photon and CARS live imaging in mice to fill that gap. The mito-ATeam and mito-roGFP-Orp1 fluorescent probes had been previously validated in vitro [36] and in vivo [2] in other systems but we also validated them in vivo in our system. We did not attempt to measure absolute values of ATP or H2O2 with each probe fluorescence in vivo because firstly, this was difficult as probes are targeted to mitochondrial matrix and secondly, it was not necessary as we aimed to compare different genotypes and conditions in particular along time. So only relative values obtained in the same imaging conditions are shown here. We found that the range of detection was large enough to cover the changes occurring in axonal mitochondria in vivo. Moreover the probe sensitivity was sufficient to observe variations in real-time using a 5 min delay between measures. This delay was partly imposed by relative low speed of our imaging system (1 min to create 10 scans of 200μm^2^ over 40 μm depth for each wavelength), but also by the intrinsic limits of our set-up: electrical stimulation induced slight contractions of saphenous nerve surrounding muscles which made the pictures blurred during the stimulation. The probes alterations were also reversible fast enough to observe changes in both directions.

Mito-ATeam was stable, but mito-roGFP-Orp1 showed a slight increase of its fluorescence ratio over time under our experimental settings. These results are not explained by photobleaching or degradation of the probe, since the total fluorescent signal does not decrease. This change in ratio of fluorescence may be caused by air oxidation [22]. However, buffered artificial cerebrospinal fluid as an incubation medium for the saphenous nerve did not prevent the probe oxidation. Nonetheless, this slow oxidation of mito-roGFP-Orp1 had negligible effects on our experiments, since it was too slow to cause significant changes in our relatively short term imaging.

Our probes gave us an instant read-out of ATP and H2O2 levels in axonal mitochondria. Since ATP export and ROS scavenging exist in mitochondria, these levels are in absolute the result of ATP and ROS production minus ATP export and ROS scavenging. However, during time-lapse imaging over a relatively short period (minutes), we considered that ATP export and ROS scavenging were unlikely to be the main players of the changes we observed. Indeed, ADP/ATP translocase, the main exporter of ATP, mediates a relative steady-state ADP/ATP exchange rate [25, 47] and ROS scavenging occurs through antioxidant enzymes that are always heavily expressed within mitochondria [27]. So, in these conditions, we believe that these fluorescent probe changes reflect mainly changes in ATP and ROS production.

Healthy mitochondria produce ATP through oxidative phosphorylation and as a consequence, ROS levels also increase. So in healthy mitochondria ATP and ROS productions are usually correlated in the same direction, meaning they are coupled. When changes in mitochondrial ATP and ROS productions are not correlated in the same direction (i.e. decreased ATP, but increased ROS or the reverse) we defined this as a decoupling. Axonal activity induced stereotypic changes in ATP and H_2_O_2_ production by axonal mitochondria. Shortly after the stimulation, production of ROS overcomes the scavenging capacities of antioxidant enzymes resulting in a peak, and then ATP increased for several minutes. This coupling was stereotypic, because it occurred almost identically after both stimulations we did. The delay between ROS production and ATP peak production may be due to the requirement for a significant proton gradient to be generated before ATP production can detected by our probe. On the level of a single mitochondrion, this delay may be extremely short but it may be significantly longer over a large population of mitochondria as we are looking at.

After the first stimulation, not all axonal mitochondria produced more ATP, suggesting that either our stimulation protocol was not mobilizing enough axons or axonal mitochondria were deactivated, similar to some ion channels. This limitation was lifted by the second stimulation. So, it could also imply that actually our ATP probe may not be sensitive enough to detect a relatively small amount of ATP and this would delay the detection of ATP production in mitochondria until the production reaches a certain level.

On the attempts we did for further stimulations, sometimes the ATP level was maintained at high range and sometimes mitochondria did not respond anymore. The reason for these variations in the long term is unknown and we did not pursue this further. However, taken together, this suggests that ATP production in axonal mitochondria is positively correlated with the level of axonal activity and can eventually reach a plateau in our conditions. Due to technical reasons we could not measure H_2_O_2_ levels in mitochondria after several stimulations. However, looking at the first two stimulations, we conclude that H_2_O_2_ production in mitochondria is also positively correlated with axonal activity but not cumulative.

It would be interesting to look further for the molecular mechanisms that allow mitochondria to detect axolemma depolarization or ion channels activity on a very short period of time (around 1 min in our detection capacities). This fast kinetic suggests the role of ions, such as calcium ions, which are mobilized during AP firing at the node of Ranvier [63], or kinases. A mechanism involving calcium has been shown to anchor mitochondria at the node of Ranvier [77], suggesting that mitochondria are associated with the axolemma there. Consistently, we found nodal mitochondria to be more metabolically active than internodal ones. However, our data following stimulations were recorded on mitochondria independently of their location on axons. As the node is much smaller than the internodes (1 μm versus 600 μm in average in the mouse nerve) the probability we recorded nodal mitochondria is extremely small. So the molecular mechanism that mobilizes mitochondria after AP firing has to exist also in internodal mitochondria.

Looking at the known origin of ROS in mitochondria, our data suggest that the H_2_O_2_ production we detected in axonal mitochondria reflects the loading of the mitochondrial matrix with protons before the production of ATP. However, since the H_2_O_2_ production drops very quickly and, contrary to the ATP production, it is not cumulative, a more complex scheme has to be taken in consideration. Indeed, H_2_O_2_ is formed by SOD enzyme from O_2_^•−^, which directly results from the ETC. Moreover, H_2_O_2_ is then changed in H_2_O and O_2_ by several reducing enzymes, such as mitochondrial GPx, so actually the amount of H_2_O_2_ we detected is the result of equilibrium between O_2_^•−^ production and SOD and reducing enzymes’ activities. As these enzymes are highly expressed in mitochondria [27], they probably do not constitute a limiting factor and the H_2_O_2_ levels we detected still reflect axonal mitochondria metabolic activation. However, H_2_O_2_ levels drop quickly, probably because reducing enzymes are removing it quickly. In addition, these enzymes’ activities change following fast post-translational modifications such as phosphorylation [75] or environmental changes [44]. While this may explain why H_2_O_2_ levels are not incremental following several stimulations, further investigation will be needed to clarify underlying mechanisms.

Several CNS and PNS neuropathies are linked to mitochondrial dysfunctions in neurons [19, 54, 67]. Our data show that the electrical activity of neurons directly regulates the physiology and the respiration of axonal mitochondria. As axonal mitochondria are the most abundant among neuronal mitochondria, this suggests that axonal mitochondria dysfunctions may severely impact neuronal physiology and survival. We evaluated this hypothesis using a couple of peripheral neuropathy mouse model, such as a model of CMT2A disease expressing mutant MFN2^R94Q^ [13]. While in vitro data had shown increased H_2_O_2_ levels [52], but no change in ATP production [6] in neuronal mitochondria of these mice, in vivo we did not measure significant differences in these parameters between resting MFN2^R94Q^ mice and control mice. A first explanation for this discrepancy is that cultured cells are living in an environment that is significantly different from the in vivo conditions. This is especially true in terms of metabolism [82]. Another explanation is that mitochondria physiology is altered, but this can only be seen when mitochondria are challenged. Environmental changes in culture may constitute this challenge, while more physiologically relevant challenges have to be found in vivo. Our electrical stimulations were physiologically meaningful challenges for axons and, similar to control mice, we observed ATP levels increasing in several axons of mutant mice, but not all, after a first nerve stimulation. However, while control mice mitochondria increased their ATP production further following a second stimulation, no increase was observed in mutant mice mitochondria. We have shown that Mitochondria Associated Membranes (MAM), which mediate interactions between the endoplasmic reticulum and mitochondria [9], are impaired in these mutant mice. So we suggest that an impaired Ca^2+^ uptake by mitochondria through MAM [1] could alter the ATP production as it has been shown that calcium is required for the TCA cycle [16]. However, mutant mice mitochondria were still able to produce a large amount of H_2_O_2_ following both stimulations, suggesting the ETC and therefore the TCA cycle are working properly. Yet this H_2_O_2_ production was completely abnormal: it was largely delayed and was sustained for over at least 10 min. As discussed before the levels of H_2_O_2_ result from an equilibrium between several parameters, so it is difficult to explain this abnormal production. Nevertheless, a relationship between mitochondrial shape and ROS has been shown in vitro [80]. It has been suggested that smaller mitochondria result in intra-mitochondrial redistribution of cytochrome c and the pro-aging molecule p66shc that acts trough ROS upregulation [20, 30]. Smaller fragmented mitochondria, such as MFN2 mutants [80], correlate with a malfunctioning antioxidant system and an increased production of ROS [20, 52]. In any case, our data show that an altered MFN2 function lead to the dysfunction of axonal mitochondria only when axons are firing. This dysfunction is strongly deleterious as mitochondria don’t produce more ATP, but show a sustained production of deleterious ROS. The functional decoupling between ATP and H_2_O_2_ production [43], occurring in the most abundant mitochondria of the neurons and in conditions of normal firing activity, is likely to be a significant cause of the neuronal dysfunction in this peripheral neuropathy.

Looking at the node of Ranvier of MFN2 mutant mice, we found that, similar to control mice, nodal mitochondria were more metabolically active than internodal ones. As MFN2 mutant mitochondria do not increase ATP production in active axons, this suggests that nodal mitochondria are intrinsically more active even in absence of firing. This is consistent with the fact that motile mitochondria slow down in the nodes of Ranvier [48] and that they have a different morphology [15]. Moreover, nodal mitochondria may participate to axonal degeneration since this process starts in nodes of Ranvier due to high ROS levels [10]. It would have been interesting to investigate changes occurring in ATP and H_2_O_2_ production of nodal mitochondria after electrical stimulation but this was impossible due to the small size of the node and the change of focus that occurs following stimulations.

The myelin sheath that covers axons appears to be an important regulator of axonal metabolism and of mitochondria physiology. Indeed in MS, demyelination alters axonal mitochondria [45, 67, 73] and we show here that nodes of Ranvier devoid of myelin house more metabolically active mitochondria. To go further, we therefore investigated the impact of the loss of myelin on axonal mitochondria. LPC was the right tool because it induced demyelination without destroying axons [3, 42, 57]. LPC-induced demyelination resulted in a decrease of ATP levels in axonal mitochondria. This decrease was strongest after 2 weeks and was reversed by 3 weeks, suggesting that ATP production within neurons is impaired until complete remyelination. This decrease was unexpected, since AP propagation in demyelinated axons is more energy demanding: the nodal machinery and the Na^+^/K^+^ ATPase are diffused all along the axolemma and all the energy-effective benefits of myelination are lost [79]. In demyelinated area of brains affected by MS, an upregulation of ETC complexes protein expression and more complexes I and IV activity [73] was observed. Moreover, demyelinated axons showed more mitochondria [45], which is consistent with a higher energy demand. Therefore, next to a defect in ATP production, an increased export rate of mitochondrial ATP by ADP/ATP translocase [47] may also participate to the decreased mitochondrial ATP levels in demyelinated axons.

Nevertheless, H2O2 levels strongly increased in axonal mitochondria of fully demyelinated axons in absence of general oxidative stress in the tissue. This decoupling of mitochondrial oxidative phosphorylation and ATP production is in accordance with previous studies that reported a decreased ATP production, together with increased ROS levels in demyelinating diseases [29]. This suggests that actually, mitochondrial ETC complexes are more active and produce more ROS, but this may not lead to more ATP production. Such a mechanism has been shown in particular conditions where the goal of mitochondrial activity is to produce heat. Uncoupling proteins such as UCP2 permeabilize the inner mitochondrial membrane to dissipate the proton gradient [60]. This may not be the sole utility of uncoupling as this process has been shown to occur in several neurodegenerative diseases [18]. However, so far we failed to detect unusual UCP2 expression in demyelinated nerves. The high production of H_2_O_2_ during demyelination is probably not without consequences as ROS can alter the function of the ATP synthase [59]. This may explain why ATP production remains weak in mitochondria of axons that are being remyelinated 2 weeks after LPC injection, despite that ROS production is back to normal. This suggests that during demyelination, axons may recruit more mitochondria [45], or use another metabolism, such as aerobic glycolysis, in order to cover their need in ATP.

While very little amount of data exists on the homeostasis of axonal mitochondria in demyelinated axons of the PNS, in the CNS, demyelination in progressive MS results in axonal mitochondria dysfunction and ROS increase [78]. The data we collected in PNS myelinated axons on ROS production by axonal mitochondria are consistent with this. This suggests that, despite the large difference that exists between PNS and CNS myelinating glia, their similar role in segregating axonal firing machinery at nodes of Ranvier is essential for mitochondria homeostasis in axons. Nevertheless, the electrical isolation of the axon and the formation of the node of Ranvier is not the only function of the myelin sheath as it also largely participates to the metabolic homeostasis of the axon through the lactate shuttle process [28, 76]. This is particularly true for the CNS myelin [28]. In addition, MS disease is significantly different from peripheral nerve diseases, in particular because of the important role played by CNS glial cells astrocytes and microglia. So, it would be essential to confirm the data we obtained in PNS myelinated axons in CNS myelinated axons in vivo, in order to definitely conclude on the alteration of ATP and ROS production by axonal mitochondria in demyelinated lesion of progressive MS.

## Conclusion

Perturbations of ATP and ROS production in mitochondria are involved in several neurodegenerative diseases. However, the dynamics of mitochondrial ATP and ROS production in healthy and diseased axons, which represent the main volume of neurons, are unknown. Combining several in vivo techniques, we characterized the dynamics of mitochondrial ATP and H_2_O_2_ production during action potential firing in living peripheral nerve axons. In mouse models of CMT2A and demyelinating neuropathies, we showed that ATP and ROS levels change in an opposite way suggesting a decoupling of their productions in mitochondria. Together, these data provide a novel insight into the role of axonal mitochondria in the production of ATP and ROS under physiological and pathological conditions.

## Additional files

Additional file 1: LPC injection induces degeneration of the myelin sheath, but not axonal degeneration. In a healthy sciatic nerve (Before injection), axons (red) are surrounded by a myelin sheath (green). This myelin sheath is severely damaged following injection of LPC into the sciatic nerve (LPC + 1w), but no sign of axonal degeneration. Injection of PBS (PBS + 1w) does not affect axon or myelin sheath physiology. Scale = 10 μm. (PDF 4147 kb)

Additional file 2: Impact of extramitochondrial ROS on mito-roGFP-Orp1 during demyelination. Upon injection of CellROX deep red into the sciatic nerve, fluorescence signal could be detected indicative of oxidative stress. No significant differences were observed between the time points of the demyelination process, nor nerves injected with PBS. In the contralateral nerves, which were not injected with LPC or PBS, a fluorescence signal was detected as well. Before CellROX injection, no fluorescence signal was observed. At the injection site, where tissue is locally damaged due to insertion of the syringe, a much stronger fluorescence signal is observed than at the region where mitochondrial H_2_O_2_ was measured (Fig. 6c). (PDF 4122 kb)

## Abbreviations

APs: Action potentials
ATP: Adenosine triphosphate
CARS: Coherent Anti-stokes Raman Scattering
CFP: Cyan fluorescent protein
CMT: Charcot-Marie-Tooth diseases
DTT: Dithiothreitol
ETC: Electron transport chain
FRET: Fluorescence resonance energy transfer
GPx: Glutathione peroxidase
H_2_O_2_: Hydrogen peroxide
LPC: Lyso-phosphatidylcholine
MAM: Mitochondria Associated Membranes
MFN2: Mitofusin2
MS: Multiple sclerosis
O_2_^•−^: Superoxide
OH•: Hydroxyl radicals
Orp1: Oxidant Receptor Peroxidase 1
PBS: Phosphate buffered saline
PNS: Peripheral nervous system
roGFPs: Redox sensitive GFPs
ROS: Reactive oxygen species
SOD: Superoxide dismutase
Trx: Thioredoxin
YFP: Yellow fluorescent protein
