# Supplementary figures and images for: In vivo real-time dynamics of ATP and ROS production in axonal mitochondria show decoupling in mouse models of peripheral neuropathies
Source: Acta Neuropathol Commun. 2019 Jun 11;7:13. doi: 10.1186/s40478-019-0740-4 (PMC6558672; doi:10.1186/s40478-019-0740-4)

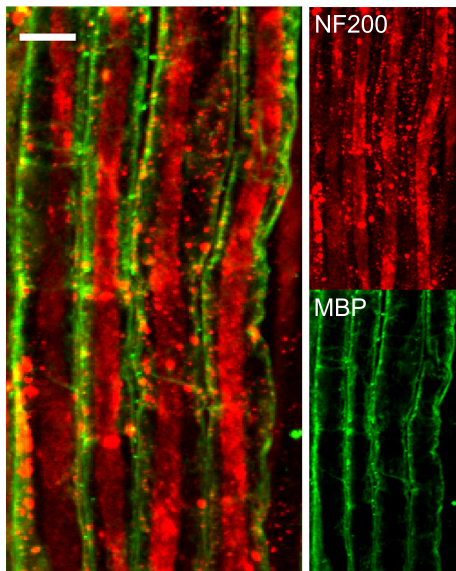

Before injection

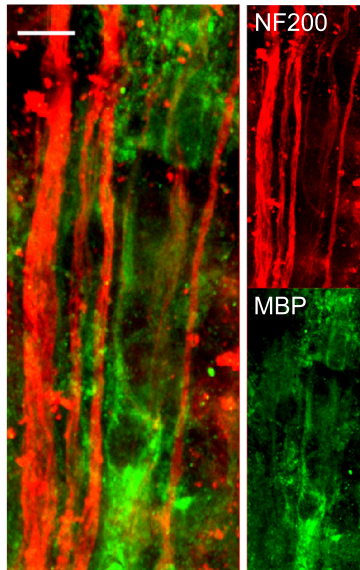

LPC + 1w

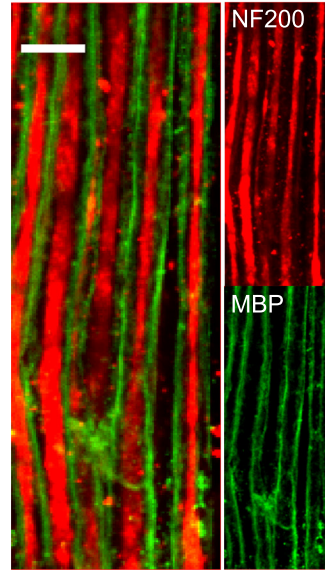

PBS + 1w

Additional File 1

Supplement: Supplementary file 1 — LPC injection induces degeneration of the myelin sheath, but not axonal degeneration. In a healthy sciatic nerve (Before injection), axons (red) are surrounded by a myelin sheath (green). This myelin sheath is severely damaged following injection of LPC into the sciatic nerve (LPC + 1w), but no sign of axonal degeneration. Injection of PBS (PBS + 1w) does not affect axon or myelin sheath physiology. Scale = 10 μm. (PDF 4147 kb) [file 40478_2019_740_MOESM1_ESM.pdf]
