# Supplementary material for: In vivo real-time dynamics of ATP and ROS production in axonal mitochondria show decoupling in mouse models of peripheral neuropathies
Source: Acta Neuropathol Commun. 2019 Jun 11;7:13. doi: 10.1186/s40478-019-0740-4 (PMC6558672; doi:10.1186/s40478-019-0740-4)
Supplement: Supplementary file 2 — Impact of extramitochondrial ROS on mito-roGFP-Orp1 during demyelination. Upon injection of CellROX deep red into the sciatic nerve, fluorescence signal could be detected indicative of oxidative stress. No significant differences were observed between the time points of the demyelination process, nor nerves injected with PBS. In the contralateral nerves, which were not injected with LPC or PBS, a fluorescence signal was detected as well. Before CellROX injection, no fluorescence signal was observed. At the injection site, where tissue is locally damaged due to insertion of the syringe, a much stronger fluorescence signal is observed than at the region where mitochondrial H2O2 was measured (Fig. 6c). (PDF 4122 kb) [file 40478_2019_740_MOESM2_ESM.pdf]

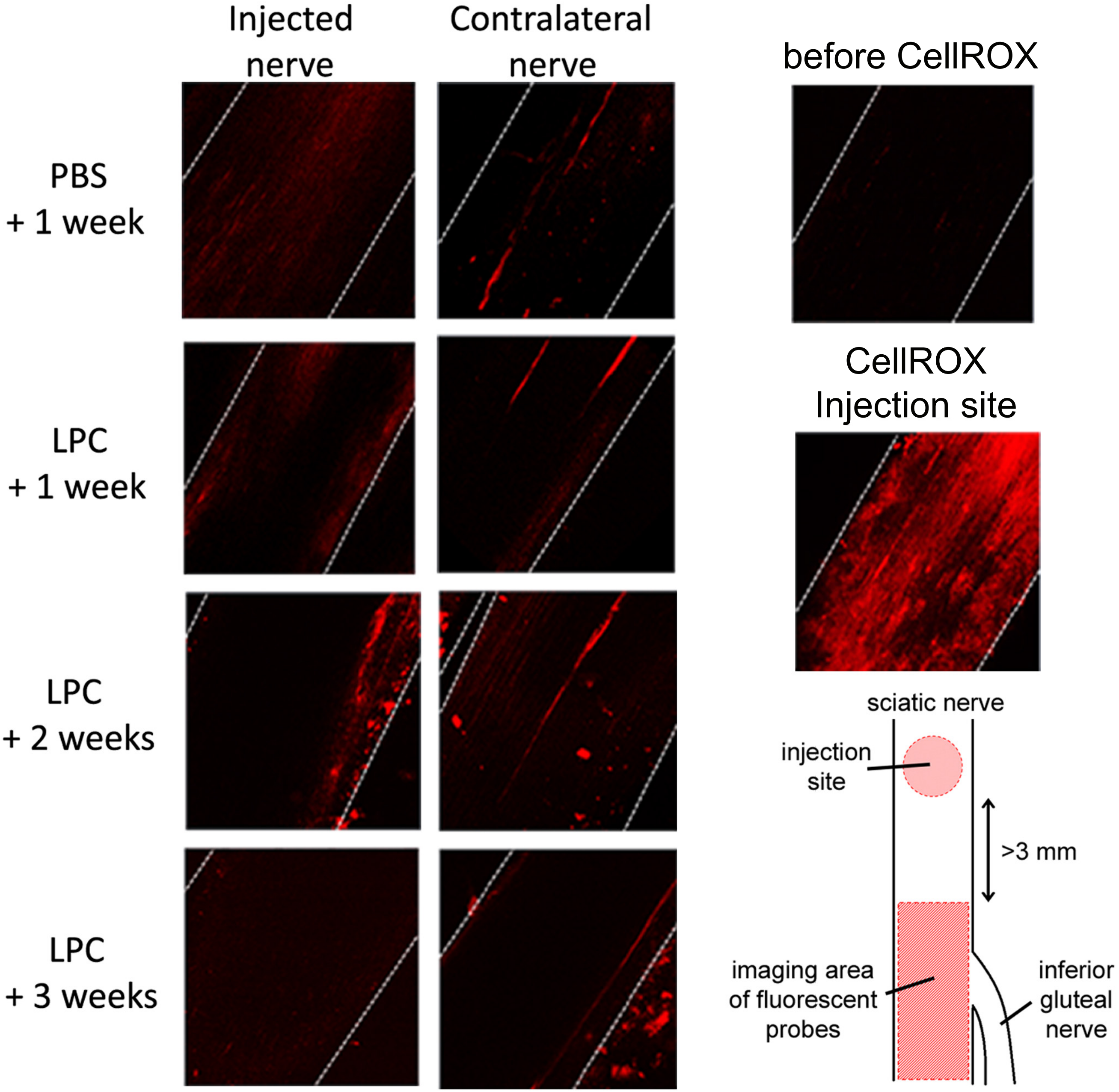

Additional File 2
